# Material matters: exploring the interplay between natural biomaterials and host immune system

**DOI:** 10.3389/fimmu.2023.1269960

**Published:** 2023-10-23

**Authors:** Alok Shiomurti Tripathi, Magdi E. A. Zaki, Sami A. Al-Hussain, Bidhyut Kumar Dubey, Prabhjot Singh, Laiba Rind, Rajnish Kumar Yadav

**Affiliations:** ^1^ Department of Pharmacology, Era College of Pharmacy, Era University, Lucknow, India; ^2^ Department of Chemistry, Faculty of Science, Imam Mohammad lbn Saud Islamic University, Riyadh, Saudi Arabia; ^3^ Department of Pharmaceutical Chemistry, Era College of Pharmacy, Era University, Lucknow, India

**Keywords:** natural biomaterials, immune response, tissue engineering, regenerative medicine, immuno-engineering

## Abstract

Biomaterials are widely used for various medical purposes, for instance, implants, tissue engineering, medical devices, and drug delivery systems. Natural biomaterials can be obtained from proteins, carbohydrates, and cell-specific sources. However, when these biomaterials are introduced into the body, they trigger an immune response which may lead to rejection and failure of the implanted device or tissue. The immune system recognizes natural biomaterials as foreign substances and triggers the activation of several immune cells, for instance, macrophages, dendritic cells, and T cells. These cells release pro-inflammatory cytokines and chemokines, which recruit other immune cells to the implantation site. The activation of the immune system can lead to an inflammatory response, which can be beneficial or detrimental, depending on the type of natural biomaterial and the extent of the immune response. These biomaterials can also influence the immune response by modulating the behavior of immune cells. For example, biomaterials with specific surface properties, such as charge and hydrophobicity, can affect the activation and differentiation of immune cells. Additionally, biomaterials can be engineered to release immunomodulatory factors, such as anti-inflammatory cytokines, to promote a tolerogenic immune response. In conclusion, the interaction between biomaterials and the body’s immune system is an intricate procedure with potential consequences for the effectiveness of therapeutics and medical devices. A better understanding of this interplay can help to design biomaterials that promote favorable immune responses and minimize adverse reactions.

## 1 Introduction

Natural biomaterials are biological materials derived from animals or plants with distinct physical and chemical characteristics that make them suitable for various biomedical applications (1). Based on their constituents and origin, they can be divided into categories. One category contains protein-based biomaterials such as collagen, fibrin, gelatin, and silk. These biomaterials are made from proteins and have unique biological characteristics. Polysaccharide-based biomaterials, such as cellulose, alginate, and chitin/chitosan, are another type. A third category includes biomaterials generated from decellularized tissues, which include decellularized heart valves, blood arteries, and the liver, among other tissues (**Table 1**) (2).

**Table 1:** Different types of natural polymer and their application.

Types	Subtypes	Example	Application
Carbohydrates Based Biomaterials	Cationic Polysaccharides	Chitosan	Mucoadhesive and Analgesic properties, Stimulate haemostasis and accelerate tissue regeneration.
	Anionic Polysaccharides	Hyaluronic acid	Cartilage, Nerve and Skin regeneration
		Alginate	Biocompatible and biodegradable nature promote wound healing.
		Heparin	Good adhesive property
	Non-ionic Polysaccharides	Cellulose	Prevent bacterial infection Hydrophilic nature
		Dextran	Protein Organisation Cell adhesion Hydrophobic surfaces
		Starch	Porous foam Polymer Self-Healing Polymer
Protein-Based Biomaterials		Collagen	Connective tissue regeneration, Triple helix conformation of collagen type-1
		Fibrin	Cell migration and Proliferation, Release cytokines and growth factors attracting cytokines
		Silk	Bone, cartilage, Liver Porous template support cell proliferation ECM Production
Decellularized Biomaterials			Closely mimics ECM architecture Mimic native ECM Bio-composition

**Figure 1:**
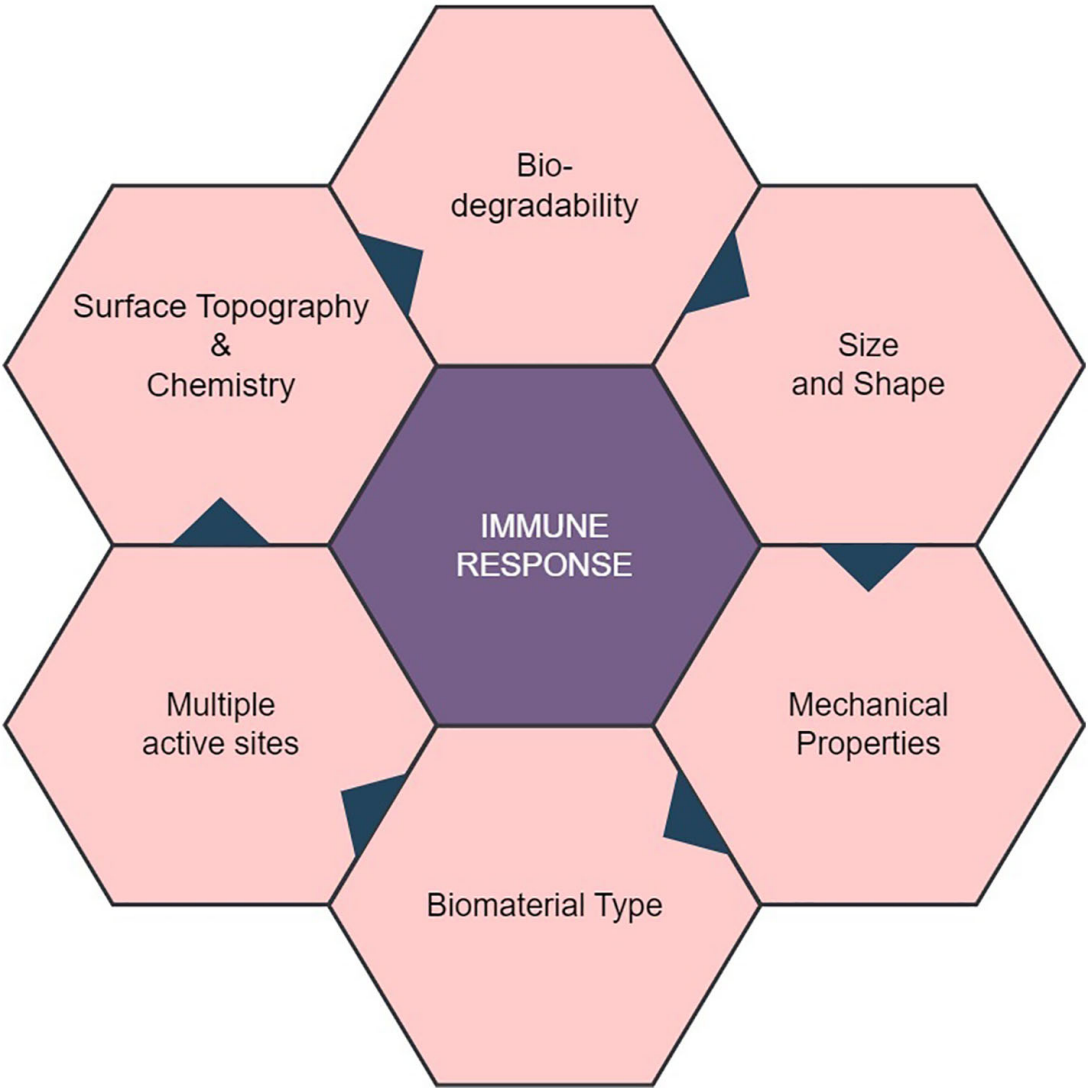
Properties of Natural Biomaterial and their Role in Interaction with Immune Response. Biocompatibility of natural biomaterial with the host’s immune system in a favorable manner, minimizing adverse reactions. Biomaterials with multiple active sites may trigger complex immune reactions, affecting their biocompatibility. The mechanical properties of the biomaterial, including stiffness and flexibility, can influence immune cell behavior and responses.

Natural biomaterials, including collagen, hyaluronic acid, and chitosan, have been widely employed in the development of medical devices, tissue engineering applications, regenerative medicine approaches, and drug delivery systems, owing to their favorable characteristics such as biocompatibility, biodegradability, and bioactivity (3). The introduction of natural biomaterials into the human body can elicit diverse immunological responses through their interaction with the immune system (**Figure 1**) (4). On the other hand, another biomaterial is synthetic biopolymer, a type of polymer that is artificially created through chemical processes and designed to mimic the properties of natural biopolymers. Polyvinyl Alcohol (PVA), Poly(ε-caprolactone) (PCL), Poly(lactic acid) (PLA), Poly(glycolic acid) (PGA), Poly(hydroxy butyrate) (PHB), and Poly(butylene succinate) (PBS) are among the examples of artificial biodegradable polymers. These polymers are intentionally synthesized through chemical processes to imitate the traits of natural biopolymers, offering properties such as biocompatibility, mechanical strength, and the ability to degrade over time (5).

The immune system is a sophisticated organized system of cells, tissues, and organs collaborating harmoniously to safeguard the body against foreign substances, including biomaterials (6). So, the question is, how does the host defense mechanism react to these natural polymers? The interplay between natural biomaterials and the body’s immune system can provide beneficial as well as adverse consequences for the efficacy of biomedical interventions (7). The innate immune system helps as the body’s primary defense counter to pathogens and foreign substances, acting as the first line of protection. It employs a range of mechanisms, including phagocytosis and cytokine release, to promptly identify and respond to potential threats (8). The interaction between natural biomaterials and the innate immune system occurs through the initiation of inflammatory responses and the activation of immune cells, particularly macrophages, which are responsible for the clearance of pathogens and debris. In contrast, the adaptive immune system is characterized by its specificity and gradual development, rendering long-lasting defense against certain infections (9). It involves T and B cells’ recognition and response to particular antigens. Natural biomaterials can also interact with the adaptive immune system by presenting antigens and promoting the stimulation and proliferation of T and B cells (10). Therefore, the interface between natural biomaterials and the immune system is critical to consider when developing biomaterials for medical applications. The impact of the immune response on the effectiveness and safety of biomaterials demands an extensive knowledge and modulation of this response. This knowledge is crucial for the advancement of successful biomaterials across various applications (11). The latest review publication, while encompassing a wide array of synthetic biodegradable polymers, notably differs from its predecessors due to its comprehensive coverage of natural biomaterials and the intricate interplay with receptor-mediated immune responses and potential adverse effects. Unlike earlier reviews that may have predominantly centered around the polymers themselves, this recent review extends its scope to delve deeply into the interaction of these materials with the body’s immune system through specific receptor.

## 2 Protein-derived biomaterials

### 2.1 Collagen

Collagen, a basic structural protein, is found in the connective tissues of animals (12). It is secreted by both fibroblasts and epithelial cells (13). Its distinct fibrillar structure offers mechanical support to various hard and soft tissues, for instance tendons, ligaments, cartilage, bone, and blood vessels (14). Till date 29 different varieties of collagen have been identified, and they all share the usual triple helix structure (15, 16). The skin, blood arteries, and internal organs all contain type III. It gives these tissues stability and support (17). The basement membrane, a thin layer of connective tissue that separates the cells of several organs, is the location where Type IV can be identified (18, 19). It supports in molecular filtration and membrane integrity preservation (20). Whereas, cell surfaces and hair contain Type V collagen (21).

Collagen can be exploited in numerous therapies, such as bone and cartilage regeneration, skin rejuvenation, and cardiovascular repair (22, 23). These different applications are made possible through distinct processing methods, such as cross-linking, complex structuring, mineralization, and carrier formation, among others. The development and use of collagen thus facilitate the advancement of tissue engineering in these various forms (24–26).

Previously, collagen was mainly considered nonimmunogenic despite some reports suggesting it may interact with antibodies and initiate antigen-antibody reactions (27, 28). Numerous cell surface receptors have been identified, exhibiting unique affinity for collagen, a prominent constituent of the extracellular matrix (ECM) found in connective tissues (29). Other biochemical signaling cascades and cellular proliferation can initiate when collagen binds to specific receptors, including immunoglobulin (Ig)-like, G6B-B, Discoidin domain receptor (DDR), osteoclast-associated receptor (OSCAR), and integrin receptors (30, 31).

#### 2.1.1 Integrin receptor pathways

Collagen is a protein with a triple-helical structure that consists of distinct amino acid sequences, including the tripeptide Gly-Pro-Hyp, which is known to interact with integrins (32, 33). The cytoplasmic tails of integrins engage in interactions with various adapter proteins, such as Src, Focal adhesion kinase (FAK), Integrin-Linked Kinase (ILK), kindlin1, paxillin, talin, vinculin, Parvin, and PINCH. These interactions act to initiate cellular signaling processes, including Akt/Phosphoinositide 3-kinase (PI3K), Protein kinase C (PKC) cascades, and Mitogen-activated Protein kinase pathways (MAPK), specifically p38, Janus Kinase (JNK), and Extracellular Signal Regulated kinase (ERK) (34, 35). Integrin-associated proteins serve a pivotal function in the activation of integrins. The mobilization and instigation of Src family kinases (SFK) by the alpha subunit of integrins serves as the initial step in beginning FAK signaling via the beta subunit. The initiation of this activation pathway results in further signaling cascades through PI3K to AKT/Protein kinase B (PKB), employing Ptdlns P3 and Src to establish focal adhesions (36–38). Following this, the activation of Rac triggers signaling via the nuclear factor kappa B (NF-κB), JNK, and p21-activated kinases (PAK) pathways. The initiation of ERK/MAPK pathways is initiated by phosphorylated FAK in the beta subunit. Furthermore, the alpha subunit exhibits the capability to activate the ERK/MAPK signaling pathway by direct initiation of SFK coupling and SHC phosphorylation, in addition to its role in FAK signaling. Research findings have demonstrated that cellular signaling pathways, which are influenced by cell adhesion, are affected by both the composition and arrangement of collagen molecules present in the ECM (39). A new study has brought attention to the distinct effects of collagen fibrils on early integrin signaling compared to monomeric collagen. Notably, collagen fibrils have been found to impede smooth muscle cell proliferation significantly (40). Moreover, a comparison between 2-D and 3-D collagen matrices has demonstrated that cell-matrix interactions are more potent in 3-D assemblies. In these 3-D environments, there is a heightened presence of selective integrin activity, leading to the stimulation of specific signaling pathways (41).

#### 2.1.2 Discoidin domain receptors pathways

In this particular biological pathway, there exist a couple of different receptor types, namely DDR1 and DDR2, which possess the ability to effectively attach to collagen molecules without requiring external activation stimuli (42). DDR1 is intricate in the alteration of multiple cellular progressions, including cell spreading, migration, adhesion, and scattering, by modulating the activity of various chemical mediators (43–46). In contrast, DDR2 is implicated in the modulation of cellular proliferation and viability through its involvement in the JNK/MAPK and PI3K/Akt signaling cascades, which exert influence on the regulation of gene expression (47, 48, 45). Following autophosphorylation, a range of subsequent signaling pathways are initiated, comprising the MAPK pathway, the PI3K pathway, and the PKC pathway (34, 49, 50). When collagen binds to DDR2, it triggers a signaling cascade that involves several chemical pathways. The JNK/MAPK pathway is activated by the phosphorylation of JNK and p38, which regulate gene expression associated with cell proliferation, differentiation, and survival (51). The PI3K/Akt pathway is also stimulated, activating Akt, which promotes cell survival by inhibiting apoptosis and regulating protein synthesis (52). Additionally, the NF-κB pathway is activated, which regulates inflammation and immune response (53). Collagen binding to DDR2 also activates RhoA, a GTPase that regulates cytoskeletal organization and cellular contractility (54). The activation of RhoA induces the phosphorylation of several cytoskeletal proteins, including myosin light chain and myosin light chain kinase. These proteins play crucial roles in cell adhesion and migration processes (55, 56).

#### 2.1.3 OSCAR-based signalling pathways

**Figure 2:**
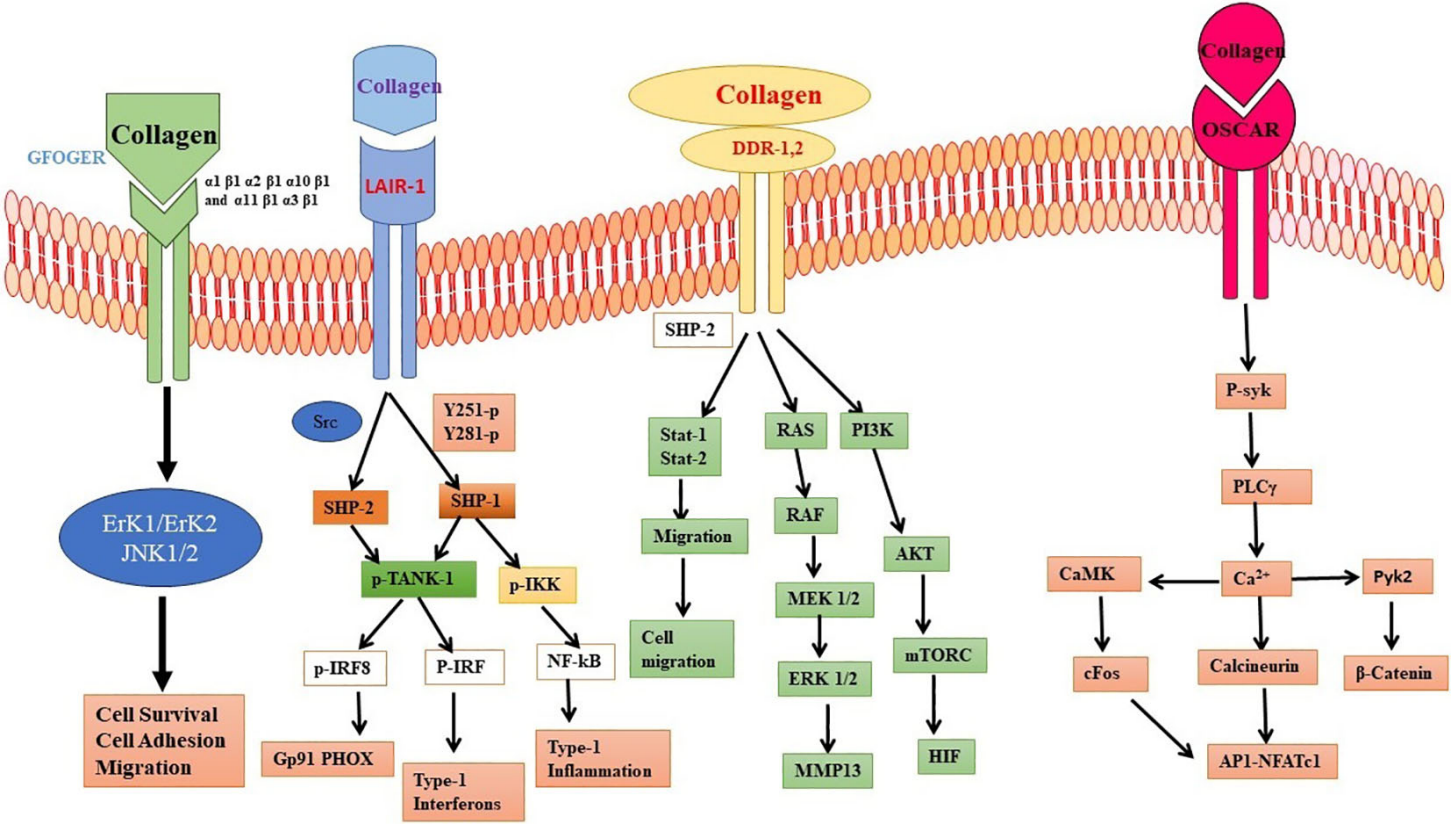
Interaction of collagen with different cellular receptor. Collagen interaction with Discoidin domain receptor (DDR), osteoclast-associated receptor (OSCAR), and integrin receptors. The interaction leads to different cellular processes involving mitogen activating protein kinases (MAPK), ERK, NFkB and cytokines.

Collagen has the ability to engage in interactions with OSCAR, which is a receptor that is present on the outer membrane of osteoclasts and dendritic cells (31, 57). One of the primary pathways started by OSCAR is the Syk/PLCγ/Ca2+ pathway, which leads to the initiation of transcription factors such as Nuclear Factor of Activated T cells 1 (NFATc1) and NF-κB and the production of various cytokines such as interleukin-6 (IL-6) and tumor necrosis factor-alpha (TNF-α) (58–63). Research findings have demonstrated that the upregulation of OSCAR is under the positive control of STAT3, which triggers the activation of the major histocompatibility complex-class II trans activator (MHC-CIITA) gene (64). On the other hand, the transcription of OSCAR is subject to negative regulation by STAT1, a process that is impeded by the presence of the protein inhibitor of activated STAT3 (PIAS3). Furthermore, interferon-gamma (IFN-γ) can induce the expression of CIITA and PIAS3, which modulates the expression of OSCAR in osteoclasts (40). When OSCAR engages with Fc receptor gamma (FcRγ), this communication activates a sequence of actions that result in the stimulation of two important signaling pathways: the calcium/calmodulin-dependent protein kinase IV (CAMK IV) pathway and the calcineurin pathway. Ultimately, this intricate process amplifies the cellular response and regulates the activation of NFATc1, which is vital for the cellular functions (65–67). This process supports osteoclast activation and maturation (68). In general, the stimulation of CAMK IV and calcineurin signaling pathways through OSCAR-FcRγ has a significant impact on enhancing the generation of NFATc1 and other transcription factors following the interaction between RANK and RANKL. This, in turn, contributes to the promotion of osteoclast activation and maturation (69, 60). Collagen binding to OSCAR activates a signaling network involving multiple pathways that regulate cellular processes, cytokine production, and cytoskeletal organization (**Figure 2** **).**

#### 2.1.4 Immunoglobulin receptors

Collagen can also interact with immunoglobulin receptors, including the FcγRs and the immunoglobulin-like transcript (ILT) receptors (70, 71). Fcγ receptors (FcγRs) are present on a variety of immune cells, such as macrophages, neutrophils, and dendritic cells. These receptors are of utmost importance in the identification and elimination of immunological complexes and infections (72, 66, 73). The engagement of FcγRs triggers the initiation of subsequent signaling cascades, which encompass the acquisition and activation of several enzymes such as tyrosine kinases, protein kinases, and phospholipases (74, 75). This activation can trigger various cellular responses such as phagocytosis, cytokine production, and antibody-dependent cell-mediated cytotoxicity (ADCC) (76, 77).

The leukocyte-associated immunoglobulin-like receptor 1 (LAIR1) is a receptor that binds to collagen and is found on multiple types of immune cells, such as B cells, T cells, natural killer (NK) cells, and dendritic cells (78). LAIR1 contains an extracellular domain that recognizes and binds to collagen and a cytoplasmic immunoreceptor tyrosine-based inhibition motif (ITIM) mediates downstream signaling (79).

When collagen binds to LAIR1, it triggers the recruitment of intracellular signaling molecules, including the tyrosine phosphatases Src homology phosphatase-1 (SHP-1) and SHP-2, which can dephosphorylate and inhibit various downstream signaling molecules (80). The process results in the dephosphorylation of spleen tyrosine kinase (Syk), zeta associated protein-70 Zap70, and PLCγ (34, 81, 82). The inhibition efficiently hinders the activation of the protein kinases including calcium signaling via the immunoreceptor tyrosine-based activation motif (ITAM) (83).

### 2.2 Gelatin

Collagen, the primary structural element of the ECM in diverse tissues, is hydrolyzed to produce gelatin, a natural protein (84). Its qualities make it useful for tissue engineering applications (85). First, gelatin may be made from various sources, containing porcine or bovine, allowing its qualities to be tailored to the demands of the different tissues (86). Additionally, the gelatin extraction procedure may be changed to produce several varieties of gelatin, such as type A or type B, which have distinct molecular weights and gelation characteristics (87). Acid hydrolyzed gelatin is classified as type A, with an isoelectric point (IP) of 5. Type B gelatin with an IP value of 9 is created by alkaline extraction (88, 89).

One of the critical advantages of gelatin is its biocompatibility, or absence of significant immunological responses or cytotoxic consequences when in contact with living cells or tissues. These materials facilitate enhanced cell adhesion, differentiation, and proliferation while being susceptible to degradation by enzymes known as metalloproteinases (90). One study claimed that shark gelatin exhibited elevated pro-inflammatory expression, it also displayed heightened levels of IL-10—an anti-inflammatory cytokine—along with increased Arginase expression, both of which are markers associated with M2-like macrophages (91). Due to its ability to be transformed into several forms, including hydrogels, films, sponges, and microspheres, gelatin is helpful in tissue engineering applications (90, 92). Gelatin constructs represent straightforward yet highly effective hydrogels designed to facilitate the controlled release of growth factors, such as BMP-2 (bone morphogenetic protein-2) or TGF-β1 (transforming growth factor beta 1), with the specific aim of promoting bone regeneration. By combining gelatin with poly(ϵ-caprolactone), a biodegradable synthetic polymer, along with a TGF-β1 inhibitor, researchers have found a way to modulate fibroblast activity. TGF-β1 is a growth factor that stimulates fibroblast proliferation and contributes to scarring. By inhibiting TGF-β1, the over-proliferation of fibroblasts can be controlled, leading to a reduction in scar formation and improved wound healing outcomes (93–95). In a different research study, a notable finding emerged when examining the effects of a specific scaffold—NT-3/fibroin coated gelatin sponge scaffold (NF-GS). This scaffold exhibited the ability to mitigate inflammation, as evidenced by a reduction in CD68 positive cells and TNF-α levels. The observation that NF-GS reduced CD68 positive cells suggests that the scaffold had a suppressive effect on macrophage activation and inflammation (96).

Gelatin also has built-in cell-adhesive motifs, such as the arginine-glycine-aspartic acid (RGD) sequence, that enable cells to cling to the biomaterial (97). These patterns encourage relationships between cells and the scaffold by allowing cells to attach to the gelatin-based biomaterial (98). Numerous extracellular matrix proteins, especially collagen, have the RGD sequence, a tripeptide sequence (99). Cell adhesion is a vital stage in tissue engineering and regenerative processes as it enables cell attachment to the biomaterial and establishes connections necessary for cellular activities, including migration, proliferation, and differentiation (100, 101).

**Figure 3:**
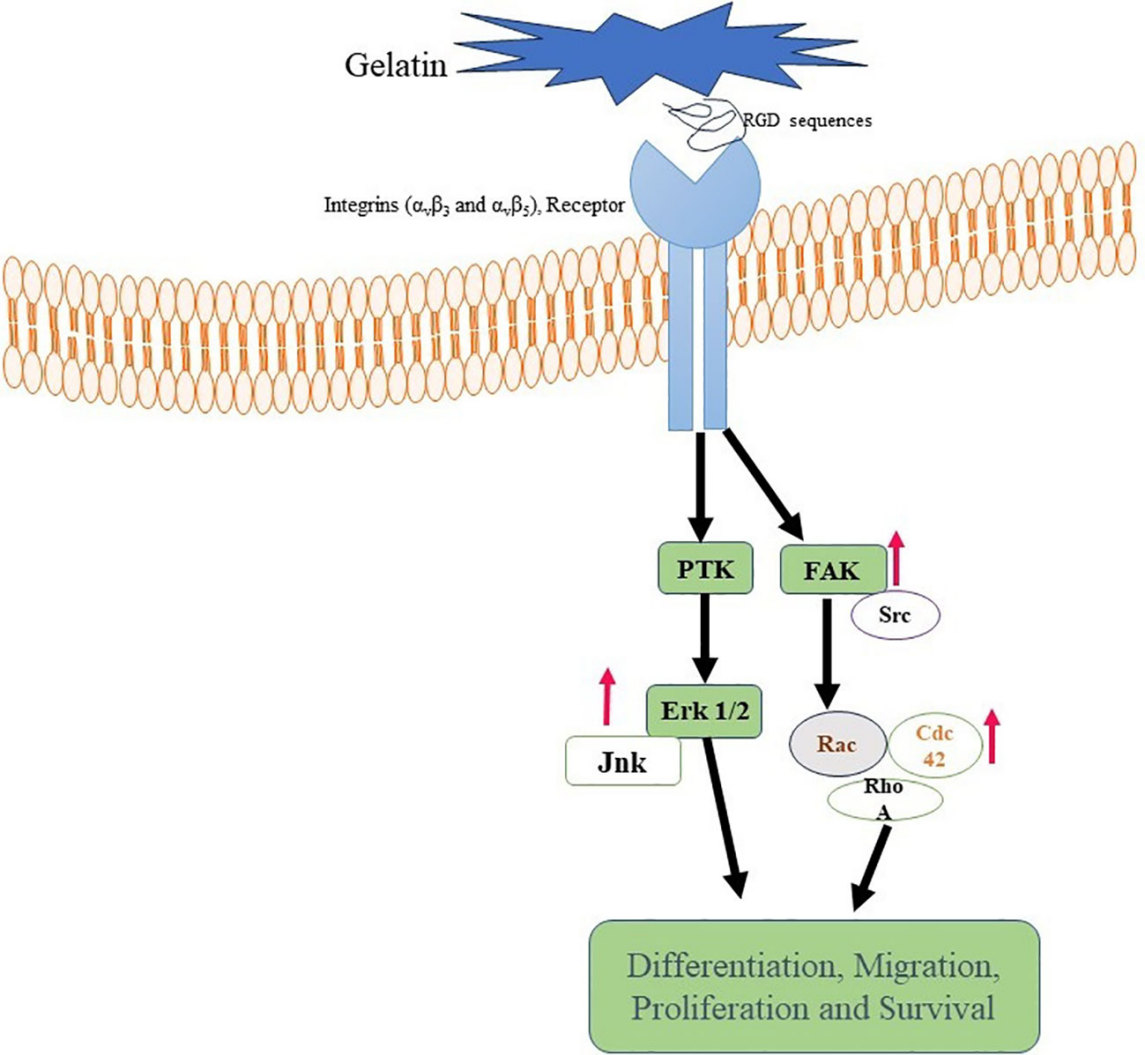
Gelatin binding with Integrin receptor. Gelatin-RGD binding with Integrin (α_v_β_3_ and α_v_β_5_) leads to activation of FAK, ErK, and Ras. These are responsible for differentiation, activation and proliferation of cells.

Specific receptors located on the cell surface, such as integrins (α_v_β_3_ and α_v_β_5_), recognize and attach to the RGD motif when cells come into contact with the RGD sequence inside the gelatin scaffold (102, 103). Gelatin’s RGD sequence may also be altered or customized to improve cell adherence and modify the substance for specific uses. For instance, the RGD motif’s density or presentation on the gelatin surface may be changed to regulate cellular responses and tweak cell adhesion (103–105) (**Figure 3**).

### 2.3 Silk

Silk has fascinated substantial consideration in tissue engineering attributable to its distinctive qualities that make it appropriate for various applications (106–109). Its biocompatibility and mechanical attributes make it appropriate for fostering tissue regeneration and aiding cell development. Silk fibroin can also be used in medicine delivery systems (110, 111). It is a desirable material for targeted medication administration since it can safely encapsulate and release medicinal ingredients (112). Due to the material’s biocompatibility and controllable breakdown, bandages that encourage wound healing while reducing scarring can be created. Silk dressings can act as a barrier of defense, control moisture levels, and promote skin cell renewal (113, 114).

It is true that the sericin component of silk fiber, in particular, has been recognized as an allergenic factor that can lead to Type I allergic symptoms, such as asthma and elevated IgE antibody levels (115). When silkworm silk fibers are exposed to sonication, soluble substances are released that accelerate the generation of pro-inflammatory cytokines and boost phagocytosis (116). It should be mentioned, though, that some researchers have shown that fibroin, the main protein found in silk, may play a part in developing Type I allergic responses (117). However, the bulk of research shows that fibroin has only a weak immune-stimulating effect (118–120).

Fibroin membranes have been discovered to stimulate macrophages less strongly than other polymers like polystyrene and poly(2-hydroxyethyl) methacrylate in the setting of macrophage activation (121). This diminished activation is related to the fact that, in contrast to the other two polymers, fibroin membranes do not adequately promote macrophage adhesion and dissemination, resulting in decreased release of the pro-inflammatory cytokine IL1 (122).

According to studies, monocytes can produce and express pro-inflammatory cytokines, including IL1b and IL6, when silk biomaterials are used to stimulate them. Nevertheless, the kind of silk biomaterial employed can affect the degree of gene expression and cytokine production (123). One research reported that the highest amounts of gene expression and cytokine production were augmented in CD14+ cells by sericin film, followed by 3D and 2D fibroin films (124). This demonstrates that sericin film has a more robust immune response than fibroin films. A separate investigation has also substantiated that both water-annealed silk film and the natural fibrous braids derived from Bombyx mori silkworms exhibited elevated levels of anti-inflammatory cytokine profiles, particularly M2 subtypes, as indicated by increased expression of IL-10, IL-13, and IL-4 (125). Differences in protein structure are likely to blame for the varied immune responses to 2D and 3D fibroin films. Comparing the three-dimensional structure of fibroin in the 3D film to the two-dimensional design in the 2D film may reveal various surface characteristics and molecular interactions (124). Variations in immunological response may result from these structural variances influencing how silk biomaterial interacts with immune cells (126).

### 2.4 Fibrin

Fibrin is a useful biomaterial extensively exercised in tissue engineering and regenerative medicine applications (127, 128). Numerous tissues, including skin, cartilage, bone, nerves, and blood vessels, have been successfully engineered using fibrin (129, 130).

Fibrin has demonstrated its potential to induce angiogenesis by binding with vascular endothelial growth factor (VEGF), which is accountable for supplying oxygen and nutrients to the cell and is commonly utilized as a component in fabricating matrices for tissue engineering (131). Additionally, fibrin has been revealed to facilitate the differentiation of transplanted mesenchymal stem cells (MSCs) into various cell types, thereby aiding in tissue regeneration (132–134).

Furthermore, fibrin and its precursor, fibrinogen play essential functions in wound healing. They control the colonization of wounds by peripheral blood mononuclear cells and macrophages, which are critical immune cells in tissue healing (135, 136). Fibrin and fibrinogen also impact the inflammatory process by boosting leukocyte (white blood cell) adhesion and influencing the production of cytokines in both leukocytes and endothelial cells, such as IL-8, IL-6, TNF-α, and reactive oxygen species (137, 138). During inflammation, these factors are involved in immune cell recruitment and activation. While fibrin and fibrinogen can influence immune responses, they may cause less inflammation and immunological response than collagen I, another widely utilized biomaterial in tissue engineering. This characteristic is advantageous as excessive inflammation can impede tissue regeneration and prolong healing (139).

Fibrinogen, a plasma protein, may bind to the Mac-1 receptor (CD11b/CD18 or M2 integrin), found on the surface of immune cells including macrophages and neutrophils (140). This connection between fibrinogen and the Mac-1 receptor is essential in controlling immune cell activities and influencing inflammatory responses (141). Several processes are involved in the biochemical function of fibrinogen binding to the Mac-1 receptor: Mac-1 is found on the cell surface in a resting state, and when activated by diverse stimuli, it undergoes conformational changes, switching to an active state (142). Mac-1 activation can be caused by various stimuli, including cytokines, chemokines, and microbial compounds (143). Mac-1 recognizes and binds to specific locations on fibrinogen when activated. The primary binding site on fibrinogen for Mac-1 is the γ-chain peptide sequence, RGD (arginine-glycine-aspartate) (144). The activated Mac-1 receptor binds to fibrinogen, explicitly engaging the RGD motif. This binding occurs via the interaction between the integrin domain of Mac-1 and the RGD sequence in fibrinogen. The binding of fibrinogen to Mac-1 triggers intracellular signaling cascades within the immune cell (145, 146, 140).

### 2.5 Elastin

Elastin is an ECM protein that provides mechanical strength. It is present in connective tissues that give flexibility and durability to different organs, including blood vessels, lungs, and skin (147–149). The hypothesis posits that the protein elastin is composed of repetitions of amino acids in the VPGXG sequence, which imparts unique rubber-like properties to the protein (150).

**Figure 4:**
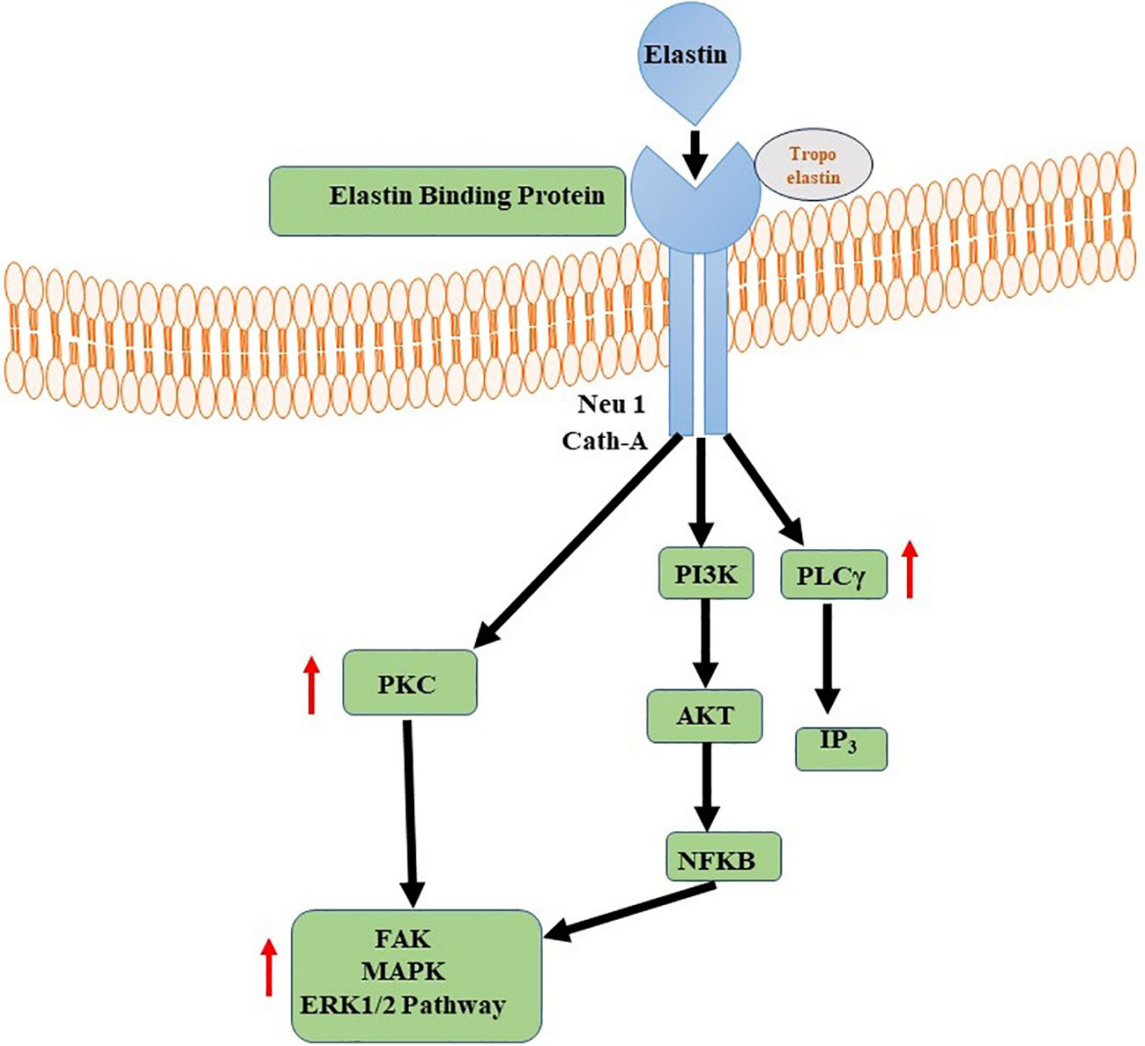
Elastin-Elastin Receptor Complexes for Immune Modulation. Phospholipase C (PLC) and Nuclear Factor Kappa B (NFKB) signalling pathways are activated by Elastin-Elastin Receptor (Elastin-ER) complexes in immunological regulation. When Elastin binds to its specialised receptor, ER, on immune cells, a series of intracellular processes occur, culminating in the activation of PLC and the production of secondary messengers. These secondary messengers activate NFKB, a transcription factor involved in the regulation of many immune-related genes.

Elastin and its derivatives have the capacity to directly engage with cells via several cell-surface receptors, such as the elastin/laminin receptor, which is a splice variation of β-galactosidase (S-Gal). The receptor in question, referred to as the elastin binding protein (EBP), has binding affinity towards the GXXPG consensus sequence (151). The EBP, a 67 kDa protein produced by several cells, aids in the construction of elastin by lining up released tropoelastin monomers and preventing premature deterioration (152). The EBP is also the principal receptor controlling gene expression, cell motility, proliferation, and differentiation. These signaling pathways may involve components of integrin signaling, FAK, MAPK pathways, PI3K/Akt pathways, among others (151, 153). The specific signaling events triggered by the elastin/laminin receptor depend on the cell type and the context of the tissue engineering environment. Elastin, along with laminin and other ECM components, contributes to the remodeling of the engineered tissue (154). The elastin/laminin receptor participates in this process by facilitating the alignment and organization of newly synthesized elastin fibers, promoting tissue maturation and functional integration (**Figure 4**) (155).

Furthermore, the design flexibility of protein-based biomaterials enables the integration of bioactive compounds, growth factors, and other medicinal agents to improve tissue regeneration processes (156). Researchers can carefully modify proteins’ structural, sequential, and other features to adapt these biomaterials to specific tissue engineering applications and accomplish desired results. There are still obstacles to achieving the best mechanical strength, stability, less immunogenic, and long-term integration of protein-based biomaterials *in vivo*. More studies are required to get over these restrictions and develop the science of protein-based tissue engineering.

## 3 Carbohydrate-based biomaterials

Carbohydrate-based biomaterials applications encompass tissue regeneration, specifically emphasizing cartilage restoration, medication delivery devices, and gel-entrapment systems designed for cell immobilization (157). These biomaterials exhibit crucial characteristics like modifiable biological activity, biodegradability, and the capacity to generate hydrogels (158, 159). What sets them apart is that they are primarily derived from natural sources. One of the critical advantages of carbohydrates is their abundance in nature, making them a sustainable and renewable resource for biomaterial development (160, 161) Researchers can tune their interactions with cells and tissues by modifying their chemical structure through functionalization or conjugation with other molecules, enabling specific biological responses. For example, introducing bioactive molecules or peptides onto polysaccharide backbones can promote cell adhesion, proliferation, and differentiation, facilitating tissue regeneration (162, 163).

Biodegradability is another significant advantage of polysaccharide-based biomaterials. Many natural polysaccharides are susceptible to enzymatic degradation, allowing them to be gradually broken down and eliminated from the body over time (164). The capability of polysaccharides to form hydrogels is also highly advantageous. Hydrogels are 3D networks accomplished with retaining higher quantity of water, and imitating the natural environment of tissues (165, 166). Polysaccharide hydrogels can be readily synthesized using physical or chemical crosslinking techniques, thereby establishing a framework conducive to cellular growth, proliferation, and tissue development (167).

Several polysaccharides, including alginate, cellulose, chitosan, and hyaluronic acid (HA), etc. are currently utilized in tissue engineering. These polysaccharides have been widely applied due to their unique properties and suitability for tissue engineering (168, 169).

### 3.1 Alginate

Structurally, Alginate is an anionic copolymer that has a linear, unbranched structure made of alternating blocks of 1,4-linked mannuronic acid and 1,4-l-guluronic acid (170). It contains repeating units of these two monomers, forming a chain-like structure. The M and G blocks give alginate unique properties and are responsible for its versatility in various applications (171). According to a study, it has been observed that alginates with a high content of M blocks exhibit greater immunogenicity compared to alginates with an increased range of G blocks (172). These M block-rich alginates have been found to increase cytokine creation at significantly higher levels, up to ten times more, compared to alginates with a higher G block content (173). It is interesting to note that pure M alginate, consisting entirely of M blocks or alginates with G-blocks alone, do not possess the ability to initiate the observed immunological activation (174). Instead, it has been observed that an immunological response requires the presence of a fraction of single G residues within a high molecular weight alginate. This fraction typically ranges from 0.05 to 0.30 of the total residues (175).

The interaction between alginate and TLR4 and TLR2 has been investigated in several studies. Both receptor are pattern recognition receptors that play a crucial role in the innate immune system by distinguishing precise molecular patterns related with pathogens (176, 177). The activation of these receptors by alginate depends on the particular structural characteristics of the alginate molecule, particularly the presence of specific fractions of single G residues within high molecular weight alginate. Studies have shown that alginate materials containing a high proportion of G residues can activate TLR4, leading to the induction of cytokines like TNF-α and IL-1β (173, 178). Additionally, alginate has been found to stimulate the expression of TLR2 and TLR4 on immune cells, such as dendritic cells and macrophages, indicating an involvement of these receptors in the recognition of alginate. The signaling pathways downstream of TLR4 and TLR2 activation lead to the generation of pro-inflammatory cytokines and introduce an immune response (179, 180).

In a study conducted on C57BL/6J (B6) mice, the viability of lymphocytes was unaffected by Alginate materials. However, compared with other alginate types, particle alginate elicited the most severe inflammatory response. This was demonstrated by an increase in the generation of cytokines that include IL-1, IL-8, TNF-α, and IFN-γ (181, 182). Furthermore, it was noted that alginates with low viscosity and particle nature exhibited heightened efficacy in stimulating dendritic cells, as indicated by the increased expression of surface markers CD40, CD86, and CD80. Furthermore, the application of particulate alginate treatment resulted in a modest increase in the levels of granulocyte colony-stimulating factor (G-CSF) in macrophages (181).

The utilization of this substance is prevalent in the field of tissue engineering due to its capacity to generate hydrogels when exposed to divalent cations, such as calcium ions. Alginate hydrogels provide a 3D milieu that facilitates the development and multiplication of cells. Another study comparing gelatin methacryloyl (GelMa) and silk fibroin-gelatin (SF-G) in bioprinted constructs, it was observed that SF-G constructs fostered higher levels of proliferation among the encapsulated human bone marrow-derived mesenchymal stem cells as opposed to the GelMA constructs. Biochemical assays, as well as assessments of gene and protein expression, clearly demonstrated the superior capability of SF-G in facilitating the formation of a fibrous collagen network and promoting chondrogenesis (183).

### 3.2 Chitosan

The subject matter has garnered considerable interest owing to its optimistic possibilities in several applications within the discipline of tissue engineering (184). Chitosan and chitin are naturally occurring compounds that can be found in the exoskeleton of crustaceans such as shrimps, crabs, and crawfish, as well as in specific fungal hyphae belonging to species such as Mucor, Rhizopus, and Absidia (185). Chitosan is classified as a linear polysaccharide consisting of d-glucosamine and N-acetyl-d-glucosamine units that are randomly dispersed. The substance in question is formed from chitin, a polysaccharide composed of N-acetyl glucosamine residues. Through a process of complete deacetylation, the N-acetyl groups are removed, resulting in the creation of N-glucosamine (186, 187). Typically, Chitosan is commonly found as a copolymer composed of (1–4)-2-acetamido-2-deoxy-β-D-glucan (N-acetyl D-glucosamine) and (1–4)-2-amino-2-deoxy-β-D-glucan (D-glucosamine) units (188).

Chitosan and chitin, as biopolymers that are not endogenous to mammals, has the capacity to elicit recognition by the immune system of mammals. Multiple chitin-binding receptors have been found in mammals, including FIBCD1, NKR-P1, and RegIIIc. Furthermore, TLR-2, dectin-1, which acts as a receptor for β-glucan and leads to the formation of T helper type 17 (Th17) cells and the recruitment of neutrophils, together with the mannose receptor, are essential in facilitating immunological responses to chitin (189, 190). FIBCD1 is a transmembrane receptor that specifically detects chitin and chitosan. The gene expression of this protein has been observed in several tissues, such as the gastrointestinal tract and lungs. Its involvement in immune cell activation and host defense mechanisms has been demonstrated (191). Upon chitosan-FIBCD1 interaction, several signaling pathways may be involved. One potential pathway is the stimulation of intracellular signaling molecules, such as protein kinases, which can lead to the phosphorylation and activation of downstream signaling proteins. These activated signaling proteins can then initiate a cascade of events that regulate immune cell activation, cytokine production, and other immune-related processes (189). The activation of FIBCD1 has the potential to augment the phagocytic capacity of certain immune cells, such as macrophages and neutrophils.

**Figure 5:**
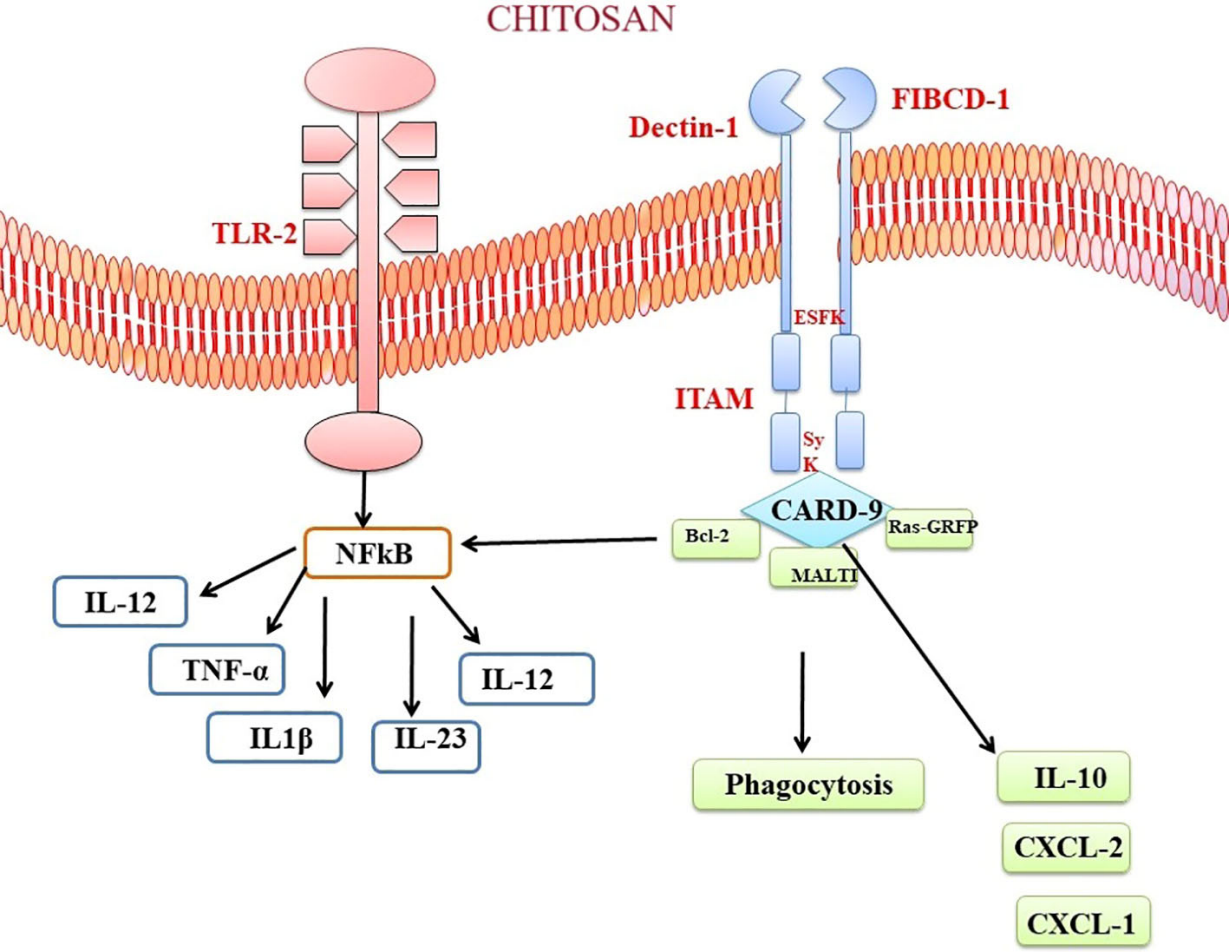
Chitosan-Mediated Immune Activation via Toll like receptor and Dectin Receptor-1 Binding. The binding of chitosan to Dectin-1 activates spleen tyrosine kinase (Syk) and caspase recruitment domain-containing protein 9 (CARD9). This complex starts a signalling cascade that stimulates the MAP kinase pathway (MALTI), which is involved in cytokine production. The anti-apoptotic protein bcl-2 can be modulated by chitosan-induced immunological activation. Chitosan’s interaction with TLR and Dectin-1 receptors modulates bcl-2 expression, which may alter cell survival and death during the immunological response.

The interplay between chitosan and TLR2 initiates signaling cascades that contribute to immune reactions and host defense mechanisms. When chitosan interacts with TLR2, it has the potential to activate many signaling pathways. The MyD88-dependent pathway is a crucial pathway. The aforementioned pathway encompasses the utilization of the adaptor protein MyD88, which subsequently triggers the activation of downstream signaling molecules, including interleukin-1 receptor-associated kinase (IRAK) and tumor necrosis factor receptor-associated factor-6 (TRAF6) (192, 189, 193). Upon contact with chitin, these molecules elicit a cascade of reactions that culminate in amplification of transcription factors, namely NF-κB and activator protein 1. The following manufacture and release of pro-inflammatory cytokines, such as IL-6, TNF-α, and IL-1β, are a result of the initiation of NF-κB and AP-1 (194). These cytokines play crucial roles in initiating and regulating the immune response, promoting inflammation, and coordinating the recruitment and activation of immune cells (**Figure 5**). Curiously, chitin exhibited the ability to expedite the process of wound healing through a pathway that relies on MyD88, and subsequently, this was succeeded by engagement with the TGF-β/Smad pathway (195). In a mouse air pouch model, chitosan sponges with 85% degree of deacetylation (DDA) that were surgically implanted within 1 and 2 days after delivery exhibited a strong attraction for numerous neutrophils and a lesser number of monocytes. On the other hand, chitosan sponges with 96% DDA attracted a comparably lower quantity of leukocytes overall, with a notable preference for monocytes over neutrophils (196). Chitosan has the potential to trigger the activation of macrophages, resulting in the secretion of nitric oxide, which could eventually lead to prolonged harm to the neighbouring tissues. Despite this aspect, chitosan has found application as biomaterial in neural therapy. To illustrate, the application of an amphipathic carboxymethyl-hexanoyl chitosan hydrogel has demonstrated the ability to enhance cell viability while preserving the gene expression reminiscent of stem cells. This is particularly pertinent in cases of induced pluripotent stem cells, a noteworthy approach for corneal reconstruction (197). Furthermore, the combination of chitosan with polylactide has been harnessed for the fabrication of fibers, which in turn have been utilized to deliver nerve growth factor to PC12 cell lines, effectively promoting nerve growth (198).

### 3.3 Cellulose

Cellulose is well recognized as the predominant and prevalent biopolymer that is present in abundance across the Earth (199). Bacterial cellulose (BC) obtained from bacteria stands out for its high purity, whereas cellulose derived from plants typically contains trace amounts of impurities like lignin, pectin, and hemicellulose (200, 201). Cellulose exhibits limited biodegradability in the human body, hence impeding the replacement of cellulose material by regenerated tissue (202, 203). Due to the structural similarity between cellulose fibers and collagen fibers found in bone tissue, cellulose has gained attention for its potential role in bone tissue engineering (203). Notably, bacterial cellulose offers a unique advantage as a localized delivery system capable of augmenting the concentration of cytokines within specific areas. Research has demonstrated that these biocompatible scaffolds are conducive to promoting osteodifferentiation, particularly when coupled with the presence of bone morphogenetic protein 2 (BMP-2) (204). A different research study documented the achievement of epithelialized skin regeneration facilitated by cellulose dressing, while also noting the absence of an inflammatory reaction. This cellulose dressing, specifically utilizing bacterial nanocellulose, demonstrated biocompatibility and found application in models involving full-thickness skin defects. The use of these porous membranes not only prompted an acceleration in the healing rate but also correlated with a reduction in the levels of inflammation (205).

Cellulose presents a possible drawback whereby it has the capacity to occupy a certain volume within the tissue that is irretrievable by the regenerating tissue (203).

The immune response to cellulose in tissue engineering applications depends on numerous features, comprising the specific type and characteristics of the cellulose-based material, its degradation properties, and the local tissue microenvironment (206). When cellulose-based biomaterials are implanted or used as scaffolds in tissue engineering, they can trigger an initial inflammatory response, similar to other biomaterials. The immediate inflammatory response is an integral component of the typical wound-healing mechanism, wherein immune cells, including neutrophils and macrophages, are recruited to the site of implantation (207). However, the extent of the immune response can be prejudiced by several aspects, containing the purity of the cellulose, the presence of impurities (such as lignin or hemicellulose), and the degree of structural modifications or functionalization of the cellulose material. The immune response to cellulose-based biomaterials can involve activating immune cells, releasing cytokines and chemokines, and remodeling of the tissue (208). Macrophages are integral components of the immune reaction to cellulose; a ubiquitous biopolymer presents in diverse natural sources. Upon meeting cellulose, macrophages exhibit distinct phenotypes, specifically the pro-inflammatory (M1) or anti-inflammatory (M2) phenotypes (209). Multiple factors contribute to the modulation of macrophage polarization. Nano cellulose has been recognized as a contributing element that influences the activation of the NLRP3 inflammasome and the generation of reactive oxygen species by macrophages. Additionally, nano cellulose has the potential to trigger anti-oxidative mechanisms within these cells. These combined effects contribute to the overall polarization of macrophages (210, 211). The polarization of macrophages can influence tissue healing, integration of the biomaterial, and tissue regeneration processes (212).

**Figure 6:**
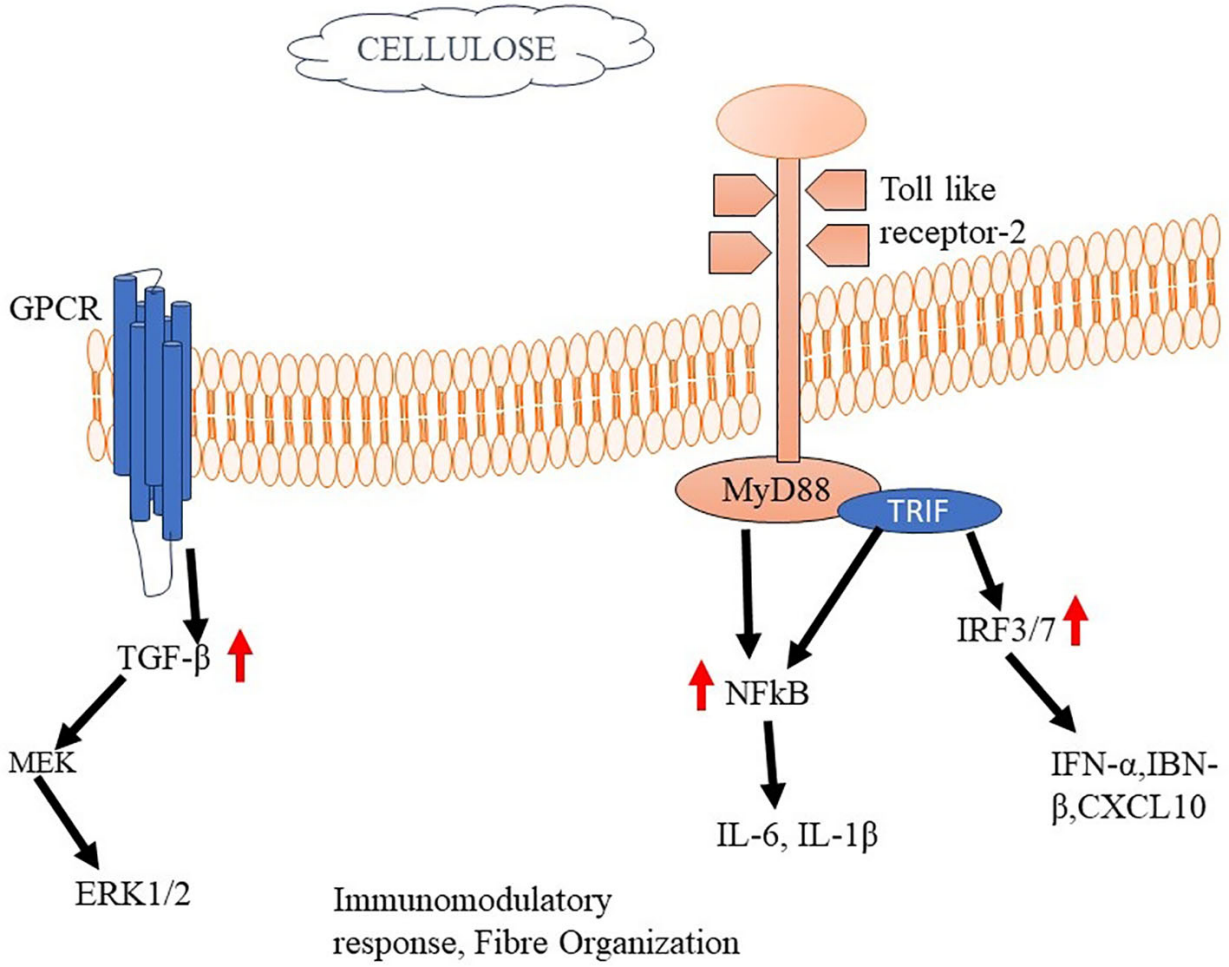
Cellulose mediated activation of Immune responses The binding of cellulose to TLR2 activates MyD88 and TRIF. Activated MyD88 serves as a scaffold for recruiting downstream signaling molecules, such as IRAK (Interleukin-1 receptor-associated kinase) and TRAF6 (TNF receptor-associated factor 6). This complex starts a signalling cascade that stimulates cytokine production and immunomodulatory responses.

The research investigation revealed that cellulose nanofibrils (CNFs) exhibited inhibitory capabilities on the growth of peripheral blood mononuclear cells (PBMCs) upon exposure to the T-cell mitogen PHA. Furthermore, it was observed that CNFs have the ability to decrease the production of interleukin-2 (IL-2) and interferon-gamma (IFN-γ), both of which are cytokines that play a crucial role in T-cell activation and the facilitation of pro-inflammatory reactions (213). The study employed THP-1 macrophages to investigate the effects of unmodified CNFs on the production of pro-inflammatory cytokines TNF-α and IL-1β. The results revealed an elevation in the levels of these cytokines in response to the presence of unmodified CNFs. Notably, the pro-inflammatory impact was not found in the cells when they were subjected to treatment with modified CNFs (214). The utilization of a crosslinking agent, namely polyethyleneimine, along with a surfactant called cetyltrimethylammonium bromide, for the purpose of modifying CNFs, led to a noteworthy diminution in both the viability and proliferation of fibroblast cells as compared to the unmodified CNFs in their pure form (215). The researchers have produced a carboxylated CNF aerogel with a porous structure, specifically designed for use in wound treatment. The observed effect of this aerogel was an enhancement in the expression of CD11b on leukocytes, as well as an augmentation in the synthesis of monocyte chemoattractant protein-1 (MCP-1/CCL). In addition, the aerogel demonstrated the capacity to decrease the concentrations of eosinophil chemotactic proteins (eotaxin/CCL11) and platelet-derived growth factor-BB (PDGF-BB) (216) (**Figure 6**).

### 3.4 Hyaluronic acid

The substance in question is a linear heteropolysaccharide that lacks sulfation, and is comprised of repeating units of disaccharides (217–219). The structure of hyaluronic acid consists of two alternating monosaccharide units: Glucuronic Acid (GlcA): This is a hexuronic acid and acts as the first monosaccharide unit in the HA repeating disaccharide. GlcA provides the negatively charged carboxyl group necessary for HA’s anionic nature. N-Acetyl-D-Glucosamine (GlcNAc) is an amino sugar and is the second monosaccharide unit in the HA repeating disaccharide (220, 221). The acetyl group contributes to HA’s hydrophobicity. The two monosaccharides are linked by a β (1→3) glycosidic bond, which means that the linkage occurs between the carbon 1 of GlcA and the carbon 3 of GlcNAc. This specific linkage pattern gives HA its characteristic linear structure (222, 223).

HA plays an essential part in tissue engineering, making it a highly required biomaterial for a extensive range of applications, due to its distinctive properties (224). HA is frequently employed as a scaffold material in the field of tissue engineering. HA-based scaffolds possess the capability to emulate the ECM of particular tissues, hence offering a conducive microenvironment for cellular growth and the formation of functional tissues (225, 226). HA has shown promising results in promoting chondrogenesis, the process of generating cartilage tissue. Its hydrophilic nature helps maintain a hydrated environment, essential for chondrocyte function and cartilage development (227, 228).

This inflammatory response can benefit tissue repair and regeneration by recruiting immune cells and promoting tissue healing. Hyaladherins refer to a class of proteins that has the ability to connect with HA and subsequently regulate its various biological functions. Several hyaladherins, such as versican and tumor necrosis factor-stimulated gene-6 (TSG-6), possess the capacity to regulate the immunological response to HA (229, 230). For instance, TSG-6 has been shown to inhibit TLR-mediated inflammation and promote tissue repair by forming a complex with HA and interacting with immune cells (231). HA has the capability to interact with CD44, a cell surface receptor expressed on human hematopoietic cells. This interaction is significant due to its potential influence on cellular behavior. A study conducted by Kajahn and colleagues unveiled that when highly sulfated HA was exposed to human monocytes, which were previously stimulated to become M1 type macrophages, it exhibited properties as an immunomodulator that supported M2 macrophage polarization. The mechanism underlying this modulation appeared to stem from a disruption in the activation signals driven by MCP-1, IL-6, and IFN-γ that typically promote M1 activation. Concurrently, the exposure to HA induced the production of IL-10—a cytokine associated with M2 macrophages—and an increase in the expression of CD163, a receptor often found on M2 macrophages. Furthermore, notable reductions were observed in the levels of IL-12 and TNFα, both of which are characteristic of M1-type macrophage responses (232).

A study examines the impact of both Methacrylated (MEHA) and maleated HA (MAHA) hydrogels on interleukin production related to inflammation using a murine macrophage cell line and BALB/c mice. The findings indicated that both MEHA and MAHA hydrogels exhibited the ability to support cell proliferation while demonstrating anti-inflammatory properties. This was evidenced by the notable increase in IL-10 level. Furthermore, the subcutaneous implantation of the materials into BALB/c mice, with observations spanning a duration of up to 28 days. Notably, the analyses from this study revealed a lack of significant chronic inflammation reaction in either the MEHA or MAHA hydrogels during the extended implantation period. This underscores the favorable long-term biocompatibility of both MEHA and MAHA hydrogels, suggesting their potential suitability for biomedical applications without causing persistent inflammation (233). The details studies of different biomaterials and immune responses are given in **Table 2**.

**Table 2:** Recent studies on interaction of Biomaterials and Immune responses.

Material Type	Disease/organ	Immune responses	Outcome	References
Collagen	Bone tissue regeneration	Phenotypic expression of osteoblast-like cells and the synthesis of the bone marker protein osteocalcin, a property specific to functional osteoblast	Bone tissue regenerates in a 3-dimensional cell culture in a type I collagen gel	(234)
Collagen Sponges	Dermal wound	Decreased time required for fibroblast migration into the wound area Improved angiogenesis	Collagen sponges containing fibroblasts or FGF exhibit increased tensile strengths and well-developed collagen networks earlier than wounds treated with a collagen sponge alone	(235)
Collagen type-1 + Neural stem cells (NSc)	Cerebral ischemia Injury	Facilitating cell migration and the delivery of oxygen and nutrients to the migrating cells	Survival of NSc engraft Synapsis formation Differentiation of Nsc	(236)
N-acetylcysteine+ collagen+ graphene oxide	Diabetic wound injury	Induce the antioxidant defense system Prompt angiogenesis and maturation of the epidermis	Promote the wound-healing process	(237)
Collagen and Hyluronic acid implant scaffold	Cancer Immunotherapy	Increase Tumor infiltration Increase the number of CD8+ T cells.	Can be a good option for payload	(238)
Alginate Hydrogels	Severe combined immunodeficient (SCID) mice	The number of blood vessels in the layer increased	Promote angiogenesis	(239)
Alginate Hydrogels	NIH 3T3 cells	Density of axon growth into the alginate hydrogel positively correlates with the diameter of channels.	Axonal regeneration	(240)
Alginate with RGD-containing peptide		Promoted osteoblast adhesion and spreading		(241)
Alginate	C57BL/6J (B6)	An increase in the generation of cytokines that include IL-1, IL-8, TNF-α, and IFN-γ	Particle alginate elicited the most severe inflammatory response	(181)
Fabricated alginate hydrogel loaded with Endostar	Colon tumor, MC-38 tumor-bearing mice	Increased proportion of CD8+ T cells in the spleen, lymph node and tumor, elevated activity of cytotoxic T lymphocytes (CTLs) and tumor cell apoptosis	Significantly reduced tumor angiogenesis	(242)
Pancreatic islets in alginate-microcapsules	Islet-xenograft	Decrease of DAMP-induced Toll-Like Receptor-2 mediated immune activation *in vitro*, and reduce peri-capsular fibrosis *in vivo* in mice	Attenuate activation of specific pro-inflammatory immune receptors locally at the transplantation site	(177)
Chitosan solution	Vaccine delivery	Enhanced antigen-specific antibody titers over five-fold and antigen-specific splenic CD4+ proliferation over six-fold.	Chitosan induced both humoral and cell-mediated immune responses	(243)
Chitosan-based core bioink	Thermal Injury	Increases in the release of wound-healing factors, epidermal growth factor, matrix metalloproteinases-9, transforming growth factor-α, platelet-derived growth factor; a decrease in pro-inflammatory factor interleukin-6, and evidence of neovascularization.	Proangiogenic, anti-inflammatory, and skin regeneration	(244)
Chitosan-enriched fibrin hydrogel	Pulpotomised rat incisors	Increase in interleukin-6 (IL-6) transcript in the Dental pulp natural killer (NK) cell population was significantly decreased. Antigen-presenting cell, myeloid dendritic cells, T cells and B cells decreased	Potential scaffold for vital-pulp therapies	(245)
Glycol-Chitosan hydrogel	Vocal fold tissue engineering	Increasing hydrogel stiffness was associated with increased anti-inflammatory.	Vocal fold	(246)
Cellulose nanocrystals	Cytotoxicity and Immunocompatibility	Unfunctionalized (uncharged) Cellulose nanocrystals form aggregates at the site of injection, inducing splenomegaly and neutrophil infiltration	The lack of an *in vitro* or *in vivo* immune response toward charged Cellulose nanocrystals for nanomaterial uses	(247)
3D Bacterial nanocellulose	Soft Tissue Implants	3D Bacterial nanocellulose did not interfere with wound haemostasis and elicited a mild acute inflammatory response, not a foreign body or chronic inflammatory response	Potential implantable biomaterial for soft tissue augmentation or replacement.	(248)

## 4 Decellularized biomaterial

Decellularized materials play a vital role in tissue engineering, offering a promising approach to create functional and biocompatible tissue constructs. The process of decellularization involves removing cellular components from donor tissues while preserving the ECM structure and bioactive molecules (249, 250). These decellularized materials serve as scaffolds that can be repopulated with recipient cells, aiding in tissue regeneration and repair. These materials can be used as hydrogels for cell encapsulation and delivery. The decellularization process can alter the composition of the ECM and thus influence the hydrogels characteristics (251). Collagen–hydroxyapatite (HA/Col) composites exemplify a remarkable class of biomaterials. These composites ingeniously combine type I collagen—a fundamental structural protein—and calcium phosphate in the apatite form, constituting the key elements of natural bone. Such synergistic combinations find practical application in the development of bone tissue substitutes. Extensive research underscores the favorable blend of biological and mechanical attributes within these biomaterials (252). The central constituents of bone, type I collagen and calcium phosphate, harmoniously intertwine within HA/Col composites. This integration not only mirrors the natural bone composition but also imparts the biomaterials with inherent biocompatibility and mechanical strength. The innate capability of these composites to interact favorably with biological systems underpins their suitability for applications in regenerative medicine. One of the standout characteristics of HA/Col composites is their versatility. They serve a dual role: as a scaffold to support the growth of nascent bone tissue and as a potential conduit for therapeutic agents (253). The scaffold function provides a 3-D framework that guides the deposition and organization of new bone material, facilitating tissue regeneration. Simultaneously, the composite material exhibits the capacity to encapsulate and deliver therapeutic compounds directly to the bone site. This unique drug delivery capability offers a strategic advantage in targeted treatments for bone-related conditions. By utilizing HA/Col composites as carriers, therapeutic agents can be released in proximity to the regenerating bone tissue, optimizing their effectiveness while minimizing systemic exposure (254). In essence, the HA/Col composites epitomize a sophisticated biomaterial platform that draws inspiration from the natural bone composition. Their harmonious blend of collagen and hydroxyapatite not only endows them with biological and mechanical prowess but also positions them as versatile tools for bone tissue engineering and targeted drug delivery strategies (255).

In a sperate study, the intriguing properties of high molecular weight hyaluronic acids (HMWHAs), which are prominent glycosaminoglycans (GAGs) within the de-cellularized extracellular matrix (dECM), come to light (256). These HMWHAs exert notable anti-inflammatory effects by interfering with the interaction between antigens and antibodies, as well as by engaging TLRs—a specific subset of pattern recognition receptors (PRRs)—and CD44 receptors located on both innate and adaptive immune cells (257). Activation of these receptors brings about a series of responses: a decline in dendritic cell (DC) maturation, a favorable shift in macrophage polarization towards the M2 phenotype, along with the differentiation of regulatory T cells and the induction of apoptosis in activated T cells. Conversely, the scenario is different for low molecular weight hyaluronic acids (LMWHAs), which are released when ECM sustains damage or undergoes degradation. These LMWHAs function as damage-associated molecular patterns (DAMPs) and set in motion inflammatory reactions. These include augmenting the chemotaxis of immune cells, maturation of DCs, and polarization of macrophages toward the M1 phenotype—once again operating through the same receptors, CD44 and TLRs. the accumulation of fragments of LMWHA within lung transplants has been highlighted as a contributing factor to chronic inflammation and the eventual rejection of the transplanted organ. The presence of these LMWHA fragments within the transplant context triggers a cascade of inflammatory responses, drawing immune cells and promoting their activation, which ultimately jeopardizes the viability and acceptance of the graft (258). An alternative study suggested that a decellularized corneal matrix cross-linked with chondroitin sulfate could potentially serve as an effective substitute for allografts and corneas sourced from human cadavers.

### 4.1 Adverse effect and clinical cases of biomaterials

The adverse effects of natural biomaterials have significant clinical implications. It’s essential for healthcare professionals, researchers, and manufacturers to carefully consider these factors when selecting, developing, and using natural biomaterials in medical applications (259). Thorough preclinical and clinical testing is necessary to assess the safety, biocompatibility, and potential risks associated with their use (260). Additionally, proper processing, sterilization, and quality control procedures are vital to mitigate adverse effects. The adverse effects of biomaterials are that to days minimised. In a European clinical trial, researchers implanted cryopreserved decellularized porcine valves into four patients. Unfortunately, three out of the four patients died within a year of the procedure. The cause of death was linked to severely inflamed and degenerated valve leaflets. The fourth patient’s valve was removed preventively shortly after implantation, likely to avoid potential complications. This outcome highlights a significant concern with the use of these decellularized porcine valves. While the concept of using animal-derived tissues that have been stripped of their cellular components (decellularized) holds promise for medical applications, these instances underscore potential challenges and risks associated with such procedures (261).

A similar, although less dramatic, situation occurred in 2012 when decellularized xenogeneic (cross-species) tissue-engineered pulmonary valve conduits were employed for reconstructing the right ventricular outflow tract in 93 patients. The term “xenogeneic” indicates that the tissue came from a different species (in this case, likely porcine or bovine sources) rather than human tissue (262). Another case study was reported that, detailing the use of a stem-cell seeded decellularized tissue-engineered tracheal graft. This intervention was performed on a compassionate basis for a young girl afflicted with critical tracheal stenosis—a severe narrowing of the trachea. Despite adhering to comprehensive Good Manufacturing Practice (GMP) protocols and employing appropriate clinical methodologies, the patient’s condition deteriorated. Unfortunately, the patient passed away three weeks after the transplantation procedure. The cause of death was attributed to an intrathoracic bleed and a sudden blockage of the airway, which led to breathing obstruction (263).

## 5 Material properties affecting immune responses

### 5.1 Surface chemistry

The effect of natural biomaterial hydrophobicity on the immunogenic response is an area of ongoing research in the field of biomaterials and immunology. Indeed, surface hydrophilicity or wettability is a significant surface property that has been found to influence the activation of anti-inflammatory macrophages and the overall immune response to biomaterials. Hydrophilic surfaces tend to resist the adsorption of proteins, which can prevent the formation of a protein corona that might trigger a pro-inflammatory response. This can lead to a more favorable microenvironment for anti-inflammatory macrophages (264, 265). A study revealed how hydrophilicity plays a pivotal role in governing the conformational adsorption of fibronectin and fibrinogen onto surfaces. This adsorption process was found to be mediated by integrin signaling, leading to subsequent activation of PI3K and NF-κB pathways (266). Another study conducted by Hotchkiss et al., demonstrated that as the surface roughness increased, indicators associated with both M1 and M2 macrophage activation exhibited an upward trend. However, when the surface roughness was combined with hydrophilicity, there was a notable suppression in the expression of pro-inflammatory markers, coupled with a significant enhancement in the expression of anti-inflammatory markers. On the other hand, the hydrophobicity of biomaterials inherently contributes to their immunogenicity, as evidenced by studies (267). Consequently, the presence of hydrophobic regions engages PRRs, initiating immune responses geared towards the elimination and resolution of perceived threats. Numerous investigations have demonstrated that biomaterial surfaces featuring -NH2 and -OH functional groups tend to provoke greater protein accumulation and immune cell migration towards the implant site, resulting in the formation of more substantial fibrous capsules around the implant, when compared to surfaces with -COOH and -CF groups (11). Barbosa et al. revealed that surfaces coated with CH3 groups demonstrated the most pronounced recruitment of Mac-1α+ phagocytes. Conversely, surfaces with OH groups, while leading to the recruitment of elevated numbers of inflammatory cells, including a substantial presence of Mac-1α+ phagocytes, exhibited the distinctive outcome of forming thinner fibrous capsules. This suggests that the nature of the surface chemistry not only influences the type and quantity of immune cells attracted to the site but also plays a role in determining the ensuing tissue response. The higher recruitment of Mac-1α+ phagocytes on CH3-covered surfaces indicates a potentially more robust immune reaction, while the formation of thinner fibrous capsules on OH-covered surfaces may imply a comparatively subdued tissue encapsulation process (268).

Surface charges are also an important parameter for immunological reaction between biomaterial and host. Studies have reported that -COOH functional groups leads to an increased negative charge across the surface. Intriguingly, this increased negative charge has been demonstrated to impede cellular growth. Furthermore, the hydroxyl group functionality (-OH) is characteristic of a neutral and hydrophilic surface. This specific arrangement is hypothesized to result in charge neutrality and a propensity for water attraction. Due to these characteristics, the -OH functionality is believed to exhibit limited affinity for proteins. Consequently, it possesses the unique property of repelling proteins, thereby contributing to an environment where protein interactions with the surface are minimized (269). While -NH2 imparts a positive charge to biomaterial surfaces, leading to enhanced adhesion, growth, and matrix formation. Specifically, fibroblasts demonstrated superior performance on -NH2 surfaces compared to alternative coatings. Notably, cells cultured on -NH2 surfaces initiated the formation of focal adhesion plaques, linked to increased cell spreading. Conversely, the introduction of –CH3 groups, which confer hydrophobic characteristics, has been demonstrated to elevate leukocyte adhesion (270). The adherence of leukocytes to solid surfaces is contingent upon a multitude of factors, encompassing surface chemistry, charge, hydrophilicity, and the process of protein adsorption (271).

### 5.2 Surface topography

Surface topography (size, shape, and texture) has been recognized as a significant factor that influences a range of cellular and physiological processes. These include vital aspects such as cell adhesion, the distribution and spreading of cells, cell motility, proliferation, differentiation, the fusion of macrophages, and the secretion of cytokines (272). Topographical features on surfaces play a fundamental role in shaping how cells interact with their surroundings, thereby impacting various cellular behaviors and physiological responses (273). Furthermore, the architectural arrangement of pore scaffolds, encompassing factors such as pore density, size, and shape, has demonstrated its influence on cellular behavior, including aspects like cell migration, proliferation, and polarization (274, 275). Moreover, this pore architecture has been found to play a role in shaping the processes of cartilage and bone formation, as well as influencing angiogenesis (276). A study reported that implanted spheres with a diameter of 1.5 mm and larger, spanning a wide range of materials such as alginate hydrogels, ceramics, metals, and plastics, notably suppressed foreign body reactions and fibrosis in comparison to smaller spheres. Furthermore, increasing sphere size led to a significant reduction of innate immune cell accumulation in peripheral tissue in C57BL/6 mice (277). An alternate study showcased that the polarization of anti-inflammatory macrophages experienced an enhancement within a limited range of roughness (Ra=0.51–1.36 μm). In cases where the roughness exceeded this range, a combination of both pro-inflammatory and anti-inflammatory markers were upregulated (278). Another study asserted that surface nanotopography results in an increased production of matrix metalloproteinase-9 from primary neutrophils (279). Study indicated that biomaterials featuring pore sizes approximately ranging from 30 to 40 mm demonstrated a correlation with the most significant influx of infiltrating macrophages. Furthermore, these biomaterials exhibited a greater proportion of M2 macrophages, along with heightened vascularization (280). These characteristics collectively contributed to optimal healing outcomes and success. Further investigation is required to better understand the relationship between biomaterial topography and immune responses in order to obtain more relevant and conclusive findings.

### 5.3 Mechanical properties

Scientific investigations have delved into the impact of biomaterial mechanical properties on immune responses, shedding light on their intricate relationship. Studies reveal that the mechanical characteristics of biomaterials, such as stiffness and elasticity, play a pivotal role in modulating immune reactions (281). Hilborn and Bjursten’s research highlighted that an elevated level of inflammation was observed in cases where a disparity existed between the stiffness of a material, the low stiffness of the surrounding tissue, and the presence of sharper edges on biomaterials with a triangular shape. This phenomenon can be attributed to an increased mechanical irritation, pointing to the role of these factors in driving the inflammatory response (282). In a recent study, it was observed that the speed of neutrophil migration was reduced on a stiff polyacrylamide (PAA) hydrogel with a stiffness of 100 kPa, as compared to a softer hydrogel with a stiffness of 5 kPa. This finding aligns with similar research and experiments that have indicated an augmented spreading of neutrophils on stiff hydrogel surfaces (283). These collective observations point toward the regulation of neutrophil extracellular traps (NETosis) – a process involving the release of neutrophil extracellular traps – by the mechanical stiffness of the substrate. The decreased migration speed of neutrophils on stiff hydrogels could potentially alter the interaction dynamics between neutrophils and the substrate, leading to enhanced neutrophil spreading (284). A recent study conducted by Sridharan et al. showcased how elevating stiffness levels influences the polarization of macrophages and shifts their migration patterns, stiffness levels resulted in amplified pro-inflammatory polarization of macrophages. Additionally, these increased stiffness levels induced a transition in the macrophages’ migration behavior (285). Equally, alternative study postulate that augmented stiffness enhances anti-inflammatory polarization through NF-κB signaling (286). Although a study demonstrated that high-stiffness responses are often influenced by Wnt/β-catenin and MAPK signaling pathways in various cell types, including macrophages, the specific signaling pathways, receptors, and transcription factors that mediate macrophage responses to biomaterial stiffness remain inadequately understood. Factors like YAP/TAZ and NF-κB have been linked to mechanotransduction and immune responses, offering potential insights into how macrophages interpret stiffness cues (287). Researchers aim to unravel the intricate molecular networks that govern macrophage behavior in order to enhance our comprehension of immune responses to biomaterials and potentially inform the design of materials for improved biocompatibility and therapeutic outcomes.

### 5.4 Biodegradation

The biodegradability of biomaterials plays a significant role in influencing immune responses. When biomaterials degrade within the body, they can release degradation products, which in turn can interact with the immune system and modulate its reactions. The relationship between biodegradability and immune responses is intricate and can vary based on factors such as the type of biomaterial, its degradation rate, and the specific immune cells involved. When silk materials are introduced into the body, they typically trigger a mild inflammatory response as part of the host’s natural reaction to foreign substances. As macrophages interact with larger silk material remnants, they might fuse together to form multinucleated giant cells known as foreign body giant cells (FBGCs) (288, 289, 115). Similar to silk proteins, the degradation rate of dECM depends on both implant-related and host-related factors. A study finds that innate immune responses to dECM involve a macrophage phenotype switch from a predominantly M1 macrophage (proinflammatory) population 3–4 days post implantation to a population enriched in M2 macrophages (anti-inflammatory and pro-healing) by 1–2 weeks following implantation. Neutrophil recruitment increased by a factor of ten after 28 days of subcutaneous implantation in mice when collagen scaffolds were cross-linked with glutaraldehyde (290). This was in stark contrast to collagen scaffolds cross-linked with hexamethylene diisocyanate, where neutrophil recruitment was lower due to the distinct degradation rates of the scaffolds. When the degradation of a scaffold occurs more rapidly than the natural regeneration and wound healing processes of the native tissue, it can result in a lack of proper ECM-like support for cells (291, 292). Consequently, the newly formed tissue might exhibit dysfunction or defects, while the by-products of degradation might not be adequately cleared from the body. On the other hand, if the degradation of the scaffold is excessively slow, it can lead to the encapsulation of the scaffold. This encapsulation can trigger an immune response from the host, ultimately causing inadequate integration or rejection by the host tissue (293, 294).

## 6 Conclusion

In conclusion, studying natural biomaterials and their interaction with the immune response holds immense promise for advancing medical science and bioengineering. Natural biomaterials offer a unique advantage over synthetic counterparts due to their inherent biocompatibility, biodegradability, and ability to promote tissue regeneration. Understanding the complicated relationship between natural biomaterials and the immune reaction is crucial for designing effective therapies and medical devices. The immune response plays a dual role in biomaterials, both as a safeguard against potential foreign invaders and a determinant of the material’s fate within the body. Researchers can devise strategies to modulate and enhance the host response to promote integration and minimize adverse reactions by comprehending how the immune system interacts with these biomaterials.

However, challenges such as immune rejection, inflammation, and foreign body reactions remain significant hurdles. As we move forward, it is imperative to prioritize safety, efficacy, and ethical considerations in developing and implementing natural biomaterial-based therapies. Rigorous preclinical and clinical studies should be performed to ensure these materials’ long-standing safety and effectiveness, paving the way for their widespread adoption in medical practice.

## Author contributions

AT: Conceptualization, Writing – review & editing. MZ: Writing – review & editing, Visualization. SA-H: Writing – review & editing, Conceptualization. BD: Writing – review & editing, Software. PS: Software, Writing – original draft. LR: Writing – original draft, Visualization. RY: Writing – original draft.

## Glossary

**Table:**

ECM	Extracellular matrix
DDR	Discoidin domain receptor
OSCAR	Osteoclast-associated receptor
FAK	Focal adhesion kinase
ILK	Integrin-Linked Kinase
PI3K	Phosphoinositide 3-kinase
MAPK	Mitogen-activated Protein kinase pathways
JNK	Janus Kinase
ERK	Extracellular Signal Regulated kinase
PKB	Protein kinase B
NF-κB	Nuclear factor kappa B
PAK	p21-activated kinases
MHC-CIITA	Major histocompatibility complex-class II trans activator
NFATc1	Nuclear Factor of Activated T cells-1
FcRγ	Fc receptor gamma
CAMK IV	Calcium/Calmodulin-dependent protein kinase IV
ILT	Immunoglobulin-like transcript receptors
LAIR1	Leukocyte-associated immunoglobulin-like receptor-1
ITIM	Immunoreceptor tyrosine-based inhibition motif
SHP-1	Src homology phosphatase-1
ADCC	Antibody-dependent cell-mediated cytotoxicity
Syk	Spleen tyrosine kinase
Zap70	Zeta associated protein-70
ITAM	Immunoreceptor tyrosine-based activation motif
IP	Isoelectric point
BMP-2	Bone morphogenetic protein-2
TGF-β1	Transforming growth factor beta-1
RGD	Arginine-glycine-aspartic acid
IL	Interleukin
VEGF	Vascular endothelial growth factor
MSCs	Mesenchymal stem cells
TNF-α	Tumor necrosis factor-α
EBP	Elastin binding protein
HA	Hyaluronic acid
TLR	Toll-Like receptor
G-CSF	Granulocyte colony-stimulating factor
GelMa	Gelatin methacryloyl
SF-G	Silk fibroin-gelatin
FIBCD-1	Fibrinogen C domain containing-1
NKR-P1	Natural killer cell surface protein P-1
IRAK	Interleukin-1 receptor-associated kinase
TRAF6	Tumor necrosis factor receptor-associated factor-6
BC	Bacterial cellulose
NLRP3	Nucleotide-binding domain, leucine-rich–containing family, pyrin domain–containing-3
CNFs	Cellulose nanofibrils
PBMCs	Peripheral blood mononuclear cells
MCP-1	Monocyte chemoattractant protein-1
PDGF-BB	Platelet-derived growth factor-BB
GlcNAc	N-Acetyl-D-Glucosamine
TSG-6	Tumor necrosis factor-stimulated gene-6
HMWHAs	High molecular weight hyaluronic acids
dECM	De-cellularized extracellular matrix
PRRs	Pattern recognition receptors
DAMPs	Damage-associated molecular patterns
NETosis	Neutrophil extracellular traps;FBGCs, Foreign body giant cells;
