# Supplementary figures and images for: Material matters: exploring the interplay between natural biomaterials and host immune system
Source: Front Immunol. 2023 Oct 23;14:1269960. doi: 10.3389/fimmu.2023.1269960 (PMC10627157; doi:10.3389/fimmu.2023.1269960)

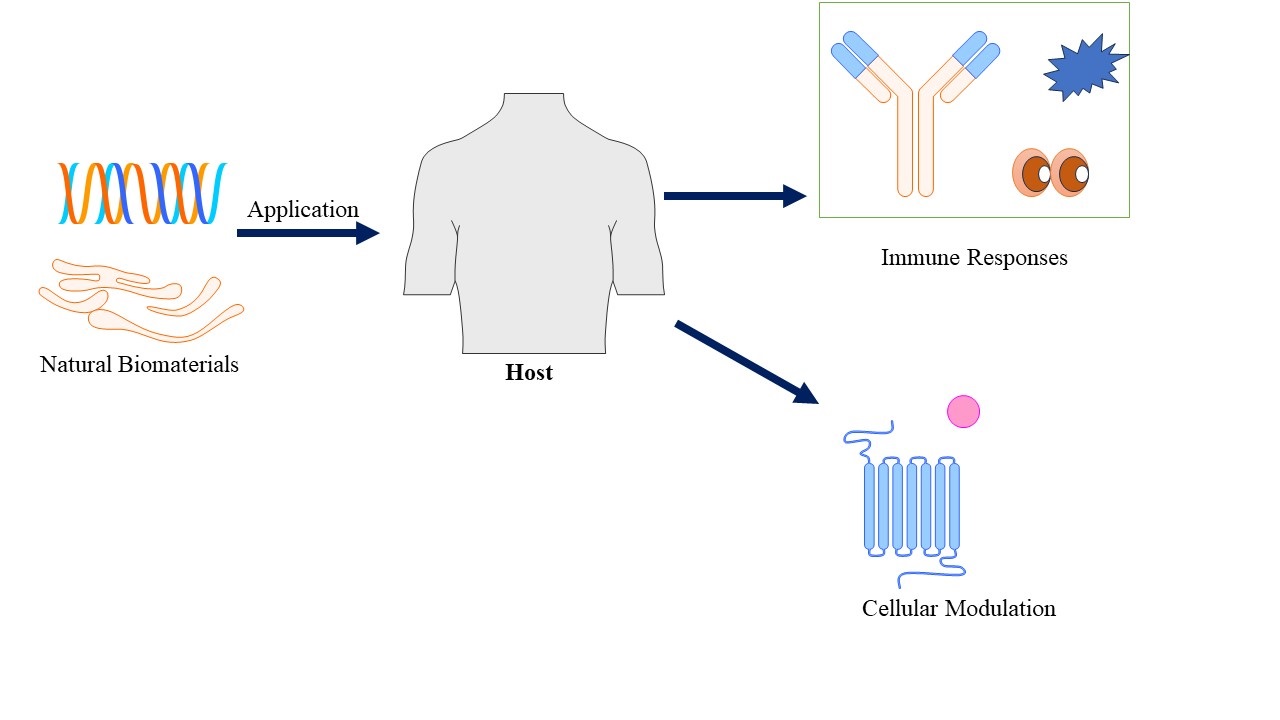

Supplement: Supplementary file 1 [file Image_1.jpg]
